# Evacuating Patients With Pediatric Cancer From Ukraine: Impact on Medical Care Capacity in Poland

**DOI:** 10.1200/GO.24.00046

**Published:** 2025-02-13

**Authors:** Aleksandra Oszer, Khrystyna Kliuchkivska, Julia Kołodrubiec, Andriy Sopilnyak, Marta Salek, Marcin W. Włodarski, Małgorzata Dutkiewicz, Zuzanna Nowicka, Maryna Krawczuk-Rybak, Jan Styczyński, Katarzyna Machnik, Ninela Irga-Jaworska, Agnieszka Mizia-Malarz, Grażyna Karolczyk, Szymon Skoczeń, Walentyna Balwierz, Katarzyna Drabko, Katarzyna Mycko, Katarzyna Derwich, Radosław Chaber, Tomasz Ociepa, Jarosław Peregud-Pogorzelski, Bożenna Dembowska-Bagińska, Paweł Łaguna, Krzysztof Kałwak, Tomasz Szczepański, Anna Raciborska, Piotr Czauderna, Asya Agulnik, Wojciech Młynarski

**Affiliations:** ^1^ Department of Pediatrics, Oncology and Hematology, Medical University of Lodz, Lodz, Poland; ^2^ Department of Pediatric Oncology, Western Ukrainian Specialized Children's Medical Center, Lviv, Ukraine; ^3^ Department of Global Pediatric Medicine, St Jude Children's Research Hospital, Memphis, TN; ^4^ Department of Hematology, St Jude Children's Research Hospital, Memphis, TN; ^5^ Fundacja Herosi, Warsaw, Poland; ^6^ Department of Biostatistics and Translational Medicine, Medical University of Lodz, Lodz, Poland; ^7^ Department of Pediatric Oncology and Hematology, Medical University of Bialystok, Bialystok, Poland; ^8^ Department of Pediatric Hematology and Oncology, Collegium Medicum, Nicolaus Copernicus University Torun, Bydgoszcz, Poland; ^9^ Unit of Pediatric Hematology and Oncology, City Hospital of Chorzow, Chorzow, Poland; ^10^ Department of Pediatrics, Hematology and Oncology, Medical University of Gdansk, Gdansk, Poland; ^11^ Department of Pediatric Oncology, Hematology and Chemotherapy, Upper Silesia Children's Care Health Centre, Medical University of Silesia, Katowice, Poland; ^12^ Department of Pediatric Oncology and Hematology, Children's Hospital in Kielce, Kielce, Poland; ^13^ Department of Pediatric Oncology and Hematology, Jagiellonian University Medical College, Cracow, Poland; ^14^ Department of Pediatric Hematology, Oncology and Transplantology, Medical University of Lublin, Lublin, Poland; ^15^ Department of Pediatrics and Hematology and Oncology, Province Children's Hospital in Olsztyn, Olsztyn, Poland; ^16^ Department of Pediatric Oncology, Hematology and Transplantology, Poznan University of Medical Sciences, Poznan, Poland; ^17^ Department of Pediatrics, Institute of Medical Sciences, Medical College, University of Rzeszow, Rzeszow, Poland; ^18^ Department of Pediatrics, Hemato-Oncology and Gastroenterology, Pomeranian Medical University, Szczecin, Poland; ^19^ Department of Pediatrics, Pediatric Oncology and Immunology, Pomeranian Medical University, Szczecin, Poland; ^20^ Department of Oncology, Children's Memorial Health Institute, Warszawa, Poland; ^21^ Department of Pediatrics Oncology, Hematology and Transplantology, Medical University of Warsaw, Warsaw, Poland; ^22^ Department of Pediatric Bone Marrow Transplantation, Oncology, and Hematology, Wroclaw Medical University, Wroclaw, Poland; ^23^ Department of Pediatric Hematology and Oncology, Zabrze, Medical University of Silesia (SUM), Katowice, Poland; ^24^ Department of Oncology and Surgical Oncology for Children and Youth, Mother and Child Institute, Warsaw, Poland; ^25^ Department of Surgery and Urology for Children and Adolescents, Medical University of Gdansk, Gdansk, Poland

## Abstract

**PURPOSE:**

Soon after the Russian invasion of Ukraine in February 2022, an initiative was organized to evacuate Ukrainian children with cancer, most initially to Poland. This study assessed the impact of this rapid increase in clinical need on the Polish system of pediatric cancer care.

**METHODS:**

This multicenter longitudinal approach was performed among all 19 Polish Pediatric Oncology Centers (PPOCs). We compared PPOC capacity before the invasion with that during the first 11 weeks after the invasion, using three ratios: patients to physicians (PtP), patients to nurses (PtN), and patients to beds (PtB). In addition, we used national data from an ongoing leukemia clinical trial to assess differences between the two study periods in the time required to comply with protocol-indicated medical procedures requiring general anesthesia.

**RESULTS:**

During the study period, 237 Ukrainian refugee children with cancer were treated in PPOCs; 60% of them arrived during the first 21 days of the war. The relative increase in patients varied significantly among the PPOCs, ranging from 42% to 460%. The average PtP, PtN, and PtB ratios increased significantly by 85%, 131%, and 105%, respectively. The portion of patients experiencing a delay in obtaining medical procedures requiring general anesthesia increased from 15% before the war to 18.2% (*P* = .043).

**CONCLUSION:**

Because of the large number of Ukrainian children with cancer were evacuated to Poland, capacity of PPOCs was reduced, affecting cancer care for all patients. Maintaining standards of pediatric oncology care in Poland would not be possible without further patient referral to medical facilities around the world by international humanitarian collaborative action.

## INTRODUCTION

Since the start of the full-scale Russian attack on Ukraine on February 24, 2022, more than 6.2 million Ukrainians have fled the country, primarily women and children. Nearly 1 million Ukrainians have sought refuge in the neighboring country of Poland.^1^ Direct assaults on major urban centers and the use of air and missile attacks throughout the country resulted in significant civilian casualties and damage to civilian infrastructure, including power plants, water supplies, and health care facilities.^2^ This destruction and fears of ongoing attacks created extraordinary challenges for the Ukrainian health care system. Not only did it need to confront the acute burden of mass casualties but also the maintenance of essential services for patients with life-threatening chronic illnesses.^3^

**CONTEXT**

**Key Objective**
How the arrival of refugee children with cancer because of invasion affects capacity of pediatric oncology centers in a neighboring country?
**Knowledge Generated**
The number of Ukrainian children with cancer admitted to Polish Pediatric Oncology Centers caused a significant increase in patients to physicians, patients to nurses, and patients to beds ratios (85%, 131%, and 105%, respectively), parameters describing reduced medical care capacity. In addition, delay in medical interventions under general anesthesia has been noticed from 15% before the war to 18.2% (*P* = .043).
**Relevance**
The relevance of this multicenter longitudinal study makes an important contribution to the knowledge of war conflicts influencing treatment of children with cancer in countries surrounded by war and emphasizes the need of international humanitarian collaborative action to sustain standards of pediatric oncology care.

Among the most vulnerable of these chronically ill patients were children with cancer.^4,5^ Recent advances in pediatric oncologic care have resulted in high cure rates and other positive outcomes. However, these benefits require compliance with proven diagnostic and therapeutic protocols that cannot tolerate substantial disruptions or delays in prescribed treatment. In response, the Polish Pediatric Oncology Centers (PPOCs) and the Polish Society of Pediatric Oncology and Hematology (PSPOH)^6^ joined with other pediatric oncology centers and organizations to establish the SAFER Ukraine initiative for the purposeful evacuation of Ukrainian children with cancer to appropriate facilities throughout Europe and North America. This system designated two centers, one in western Ukraine and the other in Poland, for the initial triage and stabilization of evacuated patients. The Polish Ministry of Health declared that Ukrainian refugee children were entitled to receive cancer treatment free of charge, similar to Polish citizens. Collaborating European governments, medical facilities, and foundations also provided requisite funds and immigration mechanisms for transportation, medical care, housing and other support services for the evacuated patients and accompanying family members. Just 2 days after the Russian invasion began, the treatment of the first Ukrainian child with cancer was arranged in Poland. The most intensive evacuation efforts were concentrated in the next 11 weeks, during which one or two convoys of approximately 100-150 patients and their families arrived at the triage center established in eastern Poland^7^ each week.

The PSPOH was responsible for examining all arriving patients and assessing their capacity for travel to a collaborating medical center in Europe or North America and arranging immediate hospitalization in Poland if necessary. This role and the rapid influx of patients generated a major challenge for the Polish system of pediatric cancer care. The PPOCs and PSPOH had previously established a centralized system of specialized pediatric oncology facilities and created a collaborative platform for multisite research, protocol implementation, and educational activities.^6^ However, the dual responsibility for conducting the initial triage assessments and managing the care of acutely hospitalized Ukrainian patients in Polish facilities placed an unprecedented burden on an already stressed Polish capacity to care for pediatric patients with cancer. This study evaluated the impact of this burden on Polish systems to continue to strengthen their capacity to provide high-quality services to both Polish and Ukrainian children with cancer and to inform a broader global discussion regarding the needs of children with catastrophic illness in areas plagued by war.^8^

## METHODS

Representatives from all PPOCs were asked to report data on Ukrainian patients treated in their centers and the baseline capacity of their departments. A standardized data collection spreadsheet was used by collaborators to record data from their centers (Data Supplement, Fig S1). All data were reviewed by investigators, and any inconsistencies were clarified with the reporting collaborators. In addition, a final data set was also cross-validated with the data collected in the SAFER Ukraine registry.^8^

### PPOC Capacity Assessment

Initially, we measured PPOC capacity using annual cancer incidence during the previous 3 years derived from the Polish pediatric oncology and hematology registry and verified locally with medical records in each PPOC. From these data, we calculated an average system volume with new diagnoses expected during an 11-week interval. Three health system load indicators were designed, considering availability of doctors, nurses, and inpatient beds in each PPOC for the expected number of new patients managed. Patients to physicians (PtP), patients to nurses (PtN), and patients to beds (PtB) ratios were calculated by dividing the number of patients treated in each institution annually by the number of physicians/nurses/beds.

### Description of the Ukrainian Patient Population

We collected data regarding Ukrainian children with oncologic and hematologic disorders admitted to the PPOC during the first 11 weeks of the war (from February 28 through May 8, 2022). This group included all individuals age 1 month to 18 years who were Ukrainian refugees treated in a member PPOC facility. We collected three types of patient data: (1) demographic and general characteristics: sex, age, Ukrainian region of residence and treatment, diagnosis, date of initial diagnosis; (2) transport and admission characteristics, including whether care was delivered as an outpatient (including clinical departments) or inpatient, date of presentation to the PPOC, type of transport (medical convoy through the SAFER Ukraine system or as individual), consultation with the SAFER Ukraine helpline; and (3) information regarding the treatment received in Polish centers (treatment interruption, change in diagnosis or treatment protocol, disease progression, relapse, death, referral to intensive care unit or palliative care, or the use of highly specialized services).

### Impact on Pediatric Oncologic Care in Poland

Before the war in Ukraine, there was documentation of limited access to complex multidisciplinary medical procedures that require general anesthesia, primarily because of a limited number of specialized medical staff. In a 2022 feasibility assessment for an interventional clinical trial (AIEOP-BFM 2017 Poland), all PPOCs reported a limited number of anesthesiologists as a barrier to protocol adherence. Thus, we used documented delays in lumbar puncture and bone marrow aspiration procedures for children treated for acute lymphoblastic leukemia because of limited access to general anesthesia to evaluate the impact of the influx of patients after February 2022 on care delivery. We used data collected by the Childhood Acute Lymphoblastic Leukemia in Poland consortium in an ongoing clinical trial AIEOP-BFM 2017, Poland (EudraCT Number: 2020-005017-41). This trial started recruitment on June 13, 2021, in all PPOCs; between June 13, 2021, and October 1, 2022, 268 children with ALL were recruited. Procedure delay was defined as the number of days between the date a medical procedure requiring general anesthesia (lumbar puncture or bone marrow biopsy) was required by the trial protocol and the date the procedure was performed. Procedure delays were compared for the periods before and after February 27, 2022, the date the first evacuated Ukrainian child with cancer was admitted to a PPOC.

### Statistical Analysis

The PtP, PtN, and PtB ratios before and after the Russian invasion of Ukraine were compared using the Wilcoxon signed-rank test. The values are presented as medians or means, according to the variable normality distribution (Shapiro-Wilk test). Chi^2^ test for 2 × 2 table was applied for frequency comparisons. *P* values of <.05 were considered significant, and all tests were two-sided. Calculations were performed using STASTISTICA 13.1 (TIBCO Software, Palo Alto, CA) and Microsoft Excel version 22.06 (Microsoft Corporation, Redmond, WA). Figures were created using Microsoft Excel via Bing, TomTom, Microsoft, and GeoNames.

The study was reviewed and approved by the human research ethics committee of the Medical University of Lodz (RNN/211/22/KE). The study was conducted in accordance with the Declaration of Helsinki and its subsequent amendments. No personal or identifiable data were included in this article.

## RESULTS

During the first 11 weeks of the war, 286 Ukrainian children were admitted to PPOCs. From these, 49 children with nononcologic diagnoses were excluded from this study. The full characteristics of the group are presented in the Data Supplement (Tables S1 and S2).

**FIG 1:**
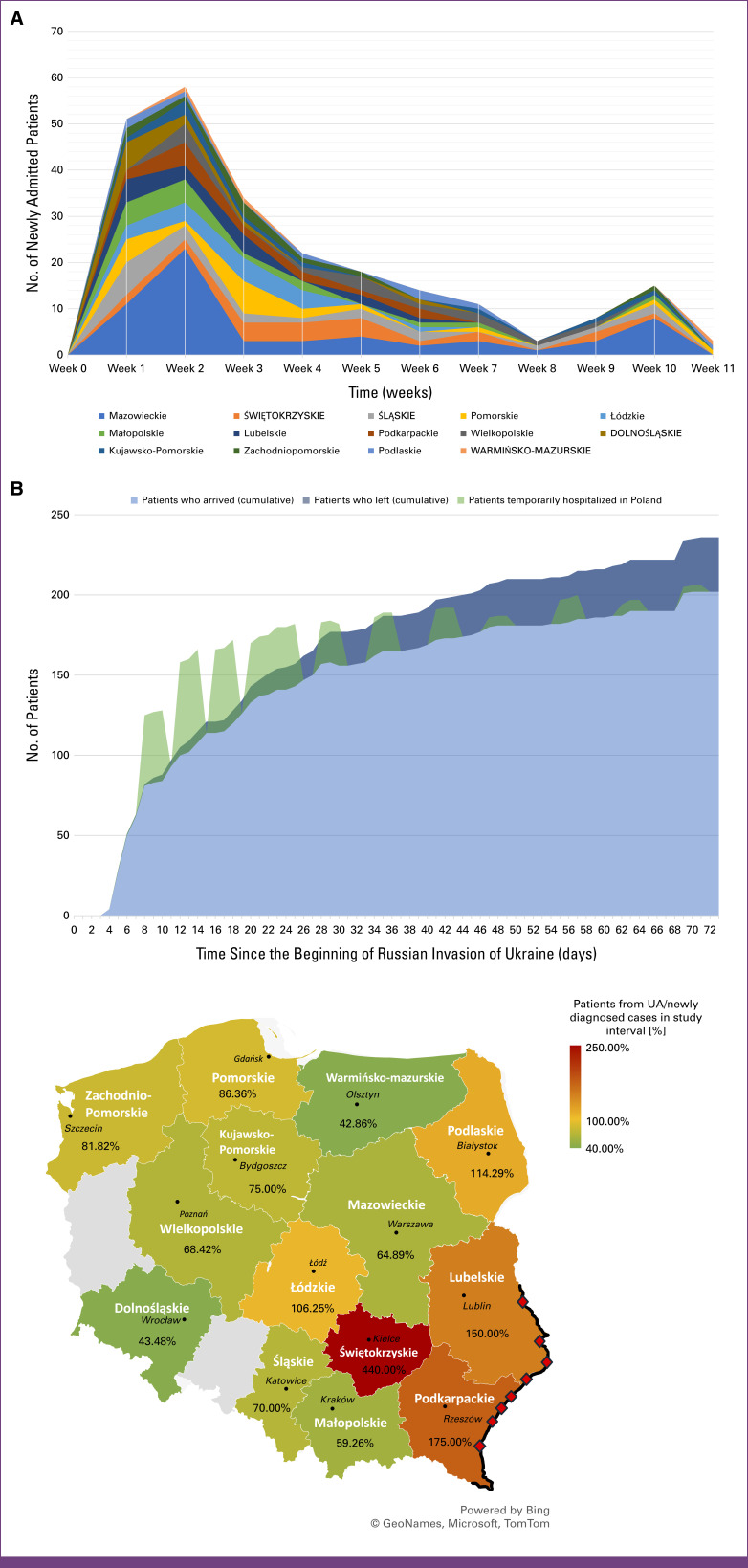
(A) Number of newly admitted patients from Ukraine, by voivodeship in Poland, on a weekly basis from the beginning of the war in Ukraine. (B) Cumulative number of patients who came from Ukraine to Polish Centers (light blue) including patients who were transferred to other country (dark blue) with the number of patients temporarily hospitalized in Poland (green) during the arranged convoys. (C) An increase in the number of patients under intensive oncologic treatment requiring periodic hospitalization presented as a percentage of the proportion of patients from Ukraine/newly diagnosed patients from Poland in each POC, after 11 weeks of the war. Red squares correspond to the crucial crossing points on the Polish-Ukrainian border. Gray zones correspond to regions with no POC. POC, Pediatric Oncology Center; UA, Ukraine.

Figures 1A and 1B shows temporal trends in patient volume over time since the start of the war. Most patients (60%) arrived in PPOCs during the first 3 weeks of the war; in the following weeks, fewer than 20 new patients arrived each week.

### Change in Medical Capacity of Polish Pediatric Oncology Centers

The number of admissions of Ukrainian children with cancer and the relative percent increase in patient volume varied considerably across the PPOC sites in 16 voivodeships (Fig 1C). The specific numbers of beds, doctors, and nurses in each PPOC are presented in the Data Supplement (Fig S2 and Table S3). The largest relative increase in patient volume was observed in the relatively small region of Świętokrzyskie, with a population of 1,178,200 inhabitants (Statistical Office in Kielce from 2022), where the increase approximated 440%. This was related to the location of the Unicorn Clinic Triage Center,^9^ from which urgent patients were transported to the closest PPOC for stabilization or hospitalization. In addition, a high relative increase was also observed in the PPOCs located close to the Ukrainian border in Rzeszów and Lublin, with 175% and 150% increases, respectively. In the Masovian Voivodeship, which has a population of 5,510,600, including Warsaw (Statistical Office in Warsaw, 2022), the increase was 65%. This relatively smaller change, despite the region's large size, is because the region already had a high number of children receiving treatment before the war. As a result, the addition of Ukrainian patients did not significantly alter the overall patient volume compared to other regions.

**FIG 2:**
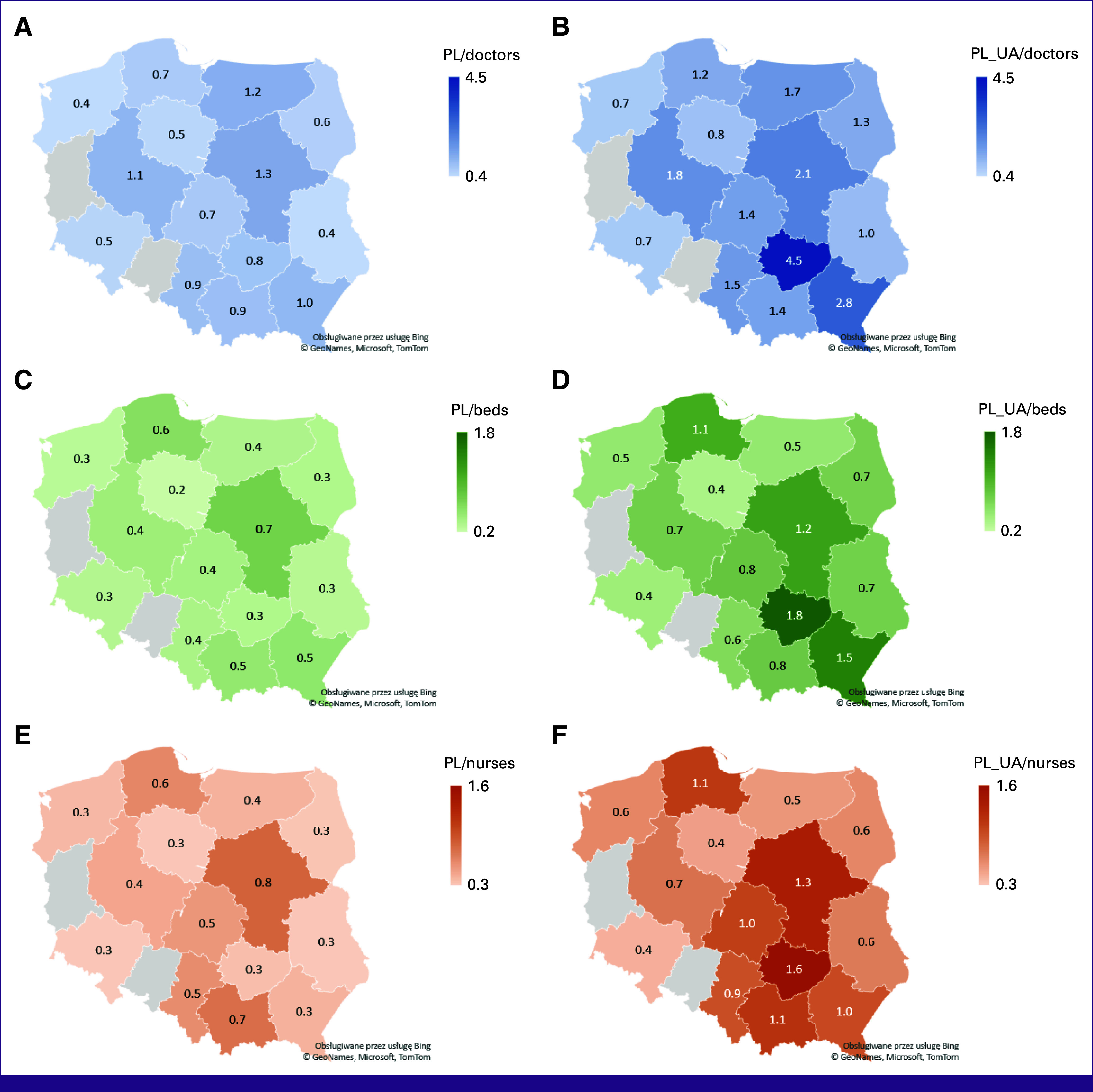
Number of Polish (A, C, and E) and Polish and Ukrainian (B, D, and F) patients per doctor (number of patients/doctor; A and B), per bed (number of patients/bed; C and D), and per nurse (number of patients/nurse; E and F), in each voivodeship in the country before and after 11 weeks of the war. PL, Poland; UA, Ukraine.

The PtP, PtN, and PtB ratios for the prewar and postinvasion study periods are presented in Figure 2. The average PtP ratio in the PPOCs was 0.76 (IQR, 0.5-1) before the invasion and 1.41 (IQR, 0.96-1.78) after the first 11 weeks postinvasion, corresponding to an increase of 85% (*P* = .001). Similarly, the PtB ratio was 0.36 (IQR, 0.31-0.5) before versus 0.74 (IQR, 0.5-1.11) after the invasion, corresponding to an increase of 131% (*P* = .001). Finally, the PtN ratio was 0.35 (IQR, 0.27-0.55) before versus 0.81 (IQR, 0.58-1.08) after the invasion, corresponding to a 105% increase (*P* = .001).

### Impact on Pediatric Oncologic Care

**FIG 3:**
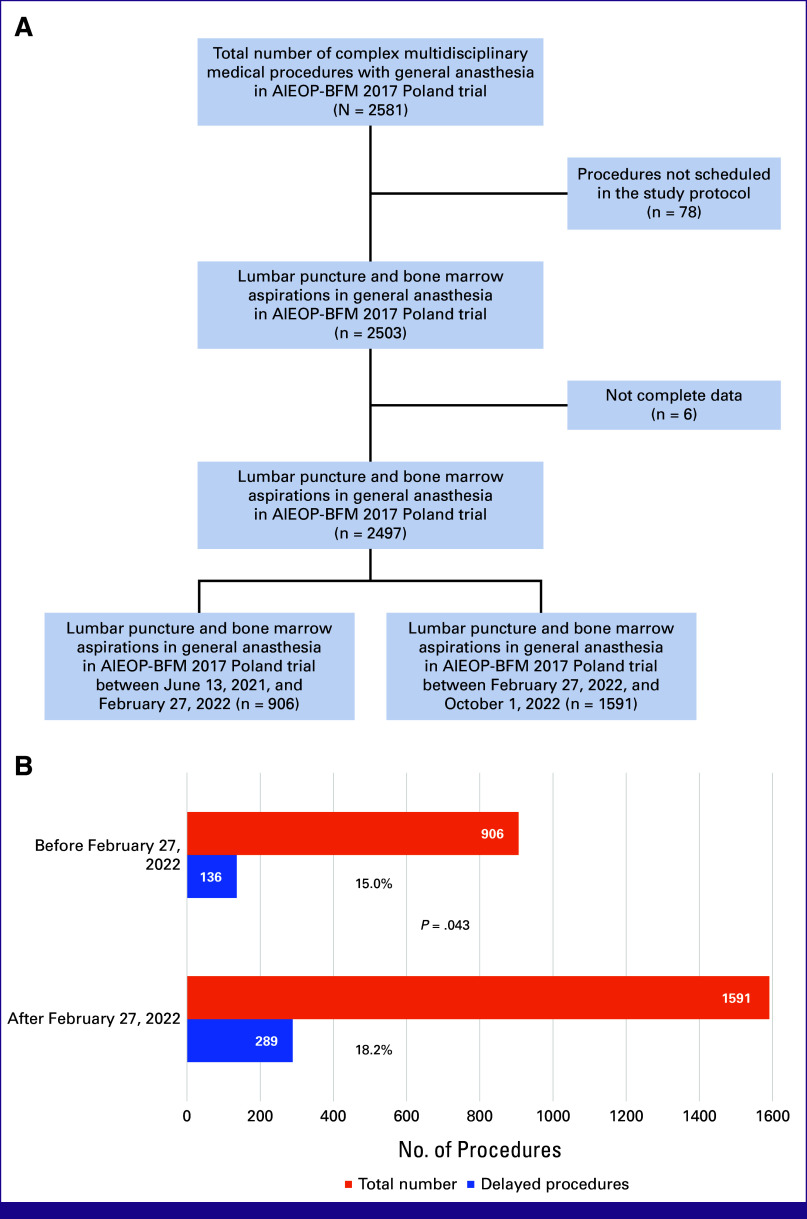
Delay in access to complex multidisciplinary medical procedures requiring general anesthesia of medical procedures identified in the ongoing AIEOP-BFM 2017 Poland trial corresponds to an impact of reduced medical capacity on medical care in pediatric oncology in Poland. (A) Structure of the complex multidisciplinary medical procedures requiring general anesthesia in eCRF of the trial; (B) frequency and numbers of delayed multidisciplinary procedures. eCRF, electronic Case Report Form.

The 268 children with ALL recruited into the AIEOP-BFM 2017 Poland trial received 2,497 medical procedures requiring general anesthesia (an average of 9.3 per patient; Fig 3B). Nine hundred six (36.3%) were performed before February 27, 2022 (Fig 3A). For 425 (17%) of these, a delay of at least 1 day was noted (mean of 1.43 days of delay). Comparing the procedures before and after the invasion, there was a significant increase in the frequency of delays, with 136 of 1,042 (15.0%) versus 289 of 1,880 (18.2%), respectively (*P* = .043). However, the number of days of delay was similar between groups (mean, 1.38 days before *v* 1.51 days postinvasion, respectively [*P* = .21]).

## DISCUSSION

Within days of the Russian invasion of Ukraine, an international network of pediatric oncologists, governments, medical institutions, and nongovernmental organizations organized a successful system for evacuating children with cancer from Ukraine.^10,11^ The greatest clinical burden of caring for the evacuated children fell to Polish physicians, nurses, medical facilities, and social support systems. This article described the nature and scope of this increased clinical challenge and the impact it had on the extant Polish pediatric oncologic system of care. Over the first 11 weeks of the war, nearly 250 Ukrainian children with cancer and blood disorders received both inpatient and outpatient care in PPOCs. This additional number of new cases presenting during a short period of time put substantial pressure on an already-strained Polish health care system.^9^ However, the relative impact varied considerably because of facility capabilities and the proximity of the clinical sites to the border and triage center locations.

While the examined system of evacuation was highly successful, this analysis suggested that the added clinical burden of patients from Ukraine on the PPOC system did result in significant increases in the ratio of patients to clinical personnel and in delays in the receipt of services for patients with ALL requiring general anesthesia. A precise understanding of the impact of this increased patient load on clinical outcomes must await the findings of an ongoing, follow-up evaluation. Nevertheless, these findings raise concerns that the Polish pediatric oncologic system might have reached a saturation point during the first 3 months of the war, a period in which full compliance with consensus standards of care might have been threatened.^11,12^

Although the challenge to the PPOCs was substantial, a more catastrophic impact was mitigated by the support provided by the collaborating partners of the larger SAFER Ukraine initiative.^8^ Only tight cooperation between the PPOCs and PSOPH with Ukrainian clinicians and essential facilitating organizations, including St Jude Global, the Tabletochki Foundation in Ukraine, The European Society for Paediatric Oncology, and Childhood Cancer International Europe, allowed for the prompt and safe evacuation of patients from Ukraine. This multidimensional, centralized response infrastructure helped protect hospital capacity in Poland by preventing an uncontrolled, continuous influx of patients and by accepting the direct transfer of patients from the Polish triage center.^7,8^ While a small number of Ukrainian patients continue to be evacuated weekly, SAFER Ukraine's primary effort has transitioned to supporting pediatric oncologic care in Ukraine and preparing for the potential impact of any escalation in the fighting in Ukraine.^13,14^

The challenge of noncommunicable, chronic conditions in humanitarian settings has attracted recent attention; however, this awareness has largely been confined to adult populations.^14^ The diverse needs of children diagnosed with serious chronic conditions, such as cancer, remain relatively unaddressed. Given the aggressive nature of pediatric cancer and the effectiveness of recent diagnostic and therapeutic advances, any substantial disruption in care may lead to disease progression and potentially preventable death.^15^ In addition, many cancers need advanced therapies, such as radiotherapy,^16^ hematopoietic stem-cell transplantation, or novel therapies that generally require sustained capabilities that are difficult or impossible to provide in areas of violent conflict. However, the ability of the systems described in this article to address these complex needs required the sustained, collaborative commitment of advanced pediatric oncologic centers throughout Europe and North America. In addition, facilitative immigration policies and adequate financial support were also essential. Soon after the Russian invasion, the Polish government moved quickly to cover the costs of treatment for Ukrainian children with cancer and blood disorders in Poland. Subsequently, all European Union countries passed similar legislation.^17^

The findings described in this report suggest potential opportunities for improving the humanitarian care provided to seriously ill children seeking refuge from war. Establishing a regional capacity to respond to humanitarian emergencies seems to be essential. This should include pragmatic planning for potential contingencies with the active engagement of regional governments, medical systems, support networks, and international humanitarian organizations that are likely to respond to a regional emergency. While this article describes an emergency response for children with cancer, there is no moral or logistical justification for not building more comprehensive capabilities to address the humanitarian needs of children with all forms of catastrophic illness. This broader commitment will require the leadership of the full range of pediatric specialty care organizations and institutions.

This study has several limitations. Our measure of treatment delays for procedures requiring anesthesia represents a single metric that should be treated only as a sentinel marker of the impact of increased patient volume on care. Further studies are also needed to evaluate the impact of the war on other regional health care systems, for children with both cancer and other complex conditions.^17,18^ In addition, our study only evaluated the short-term effects on hospital capacity and pediatric oncology treatment. Further follow-up is needed to assess the long-term effects on care provision in Poland and on the clinical and social outcomes for children and their families evacuated from Ukraine.^19,20^

In conclusion, the influx of large numbers of Ukrainian refugee children with cancer placed unprecedented stress on an already limited Polish medical capacity. There was clear evidence that the rapid increase in patient volume placed a major clinical burden on Polish medical systems and might have resulted in reduced access to services requiring skilled medical personnel. However, a more catastrophic impact was mitigated by strong support from medical institutions, professional societies, nongovernmental organizations, and governments throughout the European Union and North America. This experience underscores the indirect harm caused by war and provides some guidance as to potential opportunities to address the needs of children with catastrophic illness in other humanitarian settings around the world.

## APPENDIX. MEMBERS OF POLISH SOCIETY OF PEDIATRIC ONCOLOGY AND HEMATOLOGY AND THE SAFER UKRAINE COLLABORATIVE

Taisiya Yakimkova (St Jude Children’s Research Hospital, St Jude Global, Memphis, TN); Szymon Janczar (Department of Pediatrics, Oncology and Hematology, Medical University of Lodz, Lodz, Poland); Joanna Trelińska (Department of Pediatrics, Oncology and Hematology, Medical University of Lodz, Lodz, Poland); Łukasz Hutnik (Department of Pediatrics Hematology and Oncology, Medical University of Warsaw, Warsaw, Poland); Carlos Rodriguez-Galindo (St Jude Children’s Research Hospital, St Jude Global, Memphis, TN); Alexandra Mueller (University Medical Center, Freiburg, Germany); Paul H. Wise (Stanford University School of Medicine, Stanford, CA); Maciej Zdunek (Department of Pediatrics, Oncology and Hematology, Medical University of Lodz, Lodz, Poland).
